# Application of coagulation parameters at the time of necrotizing enterocolitis diagnosis in surgical intervention and prognosis

**DOI:** 10.1186/s12887-022-03333-y

**Published:** 2022-05-10

**Authors:** Wei Feng, Jinping Hou, Xiaohong Die, Jing Sun, Zhenhua Guo, Wei Liu, Yi Wang

**Affiliations:** Department of General & Neonatal Surgery, Children’s Hospital of Chongqing Medical University, National Clinical Research Center for Child Health and Disorders, Ministry of Education Key Laboratory of Child Development and Disorders; Chongqing Key Laboratory of Pediatrics, Chongqing, China

**Keywords:** Coagulation parameters, Coagulopathy, Necrotizing enterocolitis, Surgical intervention, Prognosis

## Abstract

**Purpose:**

It has been shown that abnormalities of coagulation and fibrinolysis system are involved in the pathogenesis of necrotizing enterocolitis (NEC), but not well studied challenge in the context of early detection of disease progression. The present study mainly explores the predictive significance of coagulation parameters at the time of NEC diagnosis in identifying the patients who eventually received surgery and/or NEC-related deaths.

**Methods:**

The retrospective study of 114 neonates with NEC was conducted with assessments of demographic data, laboratory results at the time of NEC diagnosis, treatment methods and prognosis. According to treatment methods, patients were divided into surgical intervention group and medical treatment group. Predictive factors were put forward and determined by receiver operating characteristic (ROC) curve analysis. An analysis of the surgical intervention and prognosis was performed.

**Results:**

Of 114 patients, 46 (40.4%) cases received surgical intervention and 14 (12.3%) deaths. prothrombin time (PT), PT international normalized ratio, activated partial thromboplastin time (APTT), fibrinogen and platelet count at the time of NEC diagnosis were independently associated with surgical NEC. The APTT could identify patients at high risk for surgical NEC, with 67.39% sensitivity, 86.76% specificity, better than that of other serological parameters. Coagulopathy was found in 38.6% of all patients. For surgical intervention, the area under the ROC curve (AUC) of coagulopathy was 0.869 (95% confidence interval [CI]: 0.794 ~ 0.944, *P* < 0.001), with 82.61% sensitivity and 91.18% specificity, outperformed APTT (95% CI: 0.236 ~ 0.173, *P* = 0.001). Furthermore, the AUC for coagulopathy to predict mortality was 0.809 (95% CI: 0.725 ~ 0.877, *P* < 0.001), with 92.86% sensitivity and 69.0% specificity.

**Conclusion:**

Coagulation parameters at the time of NEC diagnosis were conducive to early prediction of surgical NEC and -related deaths, which should be closely monitored in neonates at high risk of NEC and validated as a clinical decision-making tool.

## Introduction

Necrotizing enterocolitis (NEC) is one of the most common and critical gastrointestinal emergencies in neonates [1]. It is characterized by acute onset, rapid development, many complications and high mortality. Treatment during the early stages is usually medical treatment, including fasting and careful use of antibiotics, but surgery is required if pneumoperitoneum and intestinal perforation occur [2]. Currently, more than 30% of neonates with NEC may require surgery, which poses potentially devastating condition, especially the premature [3]. It has been shown that mortality rate among surgical NEC cases is estimated to be 20% ~ 30%, the highest rate among neonates requiring surgery, mandating the development of non/min-invasive and reliable biomarkers to identify validated early predictors for surgical NEC [1]. Early predictors for surgical NEC would optimize referral and treatment pathways (surgical intervention or medical treatment), and potentially lead to improve prognosis. Of course, this is also conducive to our full communication with the family members, as well as target limited medical resources for those at the highest risk.

Accumulating clinical and basic research evidence support a complex interplay between coagulation and inflammation [4–6]. Numerous studies have revealed that excessive activation of coagulation occurs in the setting of inflammation. In fact, activation of one system between coagulation and inflammation may amplify activation of the other, a situation that, if unopposed, may result in tissue damage or even multiorgan failure [5]. The role of coagulation parameters in predicting a critical prognosis of inflammatory disease (eg, sepsis, acute lung injury and appendicitis) has been described extensively in pediatrics [6–9]. More recently, the relationship between coagulation function and severity, prognosis of COVID-19 patients has been reported in many studies [10–14]. Research has revealed that coagulation dysfunction is associated with excessive inflammation, higher mortality, organ failure and prolonged hospital stay in COVID-19 patients and should be carefully addressed in clinical practice. On the other hand, although abnormalities of coagulation and fibrinolysis system are involved in the pathogenesis of NEC, hematological abnormalities related to NEC were rarely reported and the results remained controversial [15–18]. Moreover, there is a paucity of studies that specifically explore early predictors of surgical NEC. Consequently, the predictive value of coagulation parameters in identifying surgical NEC is currently of interest.

There is no study available that investigates the application of coagulation parameters in early prediction of surgical NEC and prognosis. The present study mainly explores the predictive significance of coagulation parameters in identifying the patients who eventually received surgery in a tertiary-level referral hospital, focussing on the cases got coagulation assessment at the time of NEC diagnosis.

## Materials and methods

### Study population

All procedures performed in this study involving human participants were in accordance with the relevant guidelines and regulations approved by the Institutional Research Ethics Board of Children’s Hospital of Chongqing Medical University (Date: 09.28.2021/No: 329). We completed a retrospective files review of all patients who had been diagnosed with NEC (Bell’s stage ≥ II) in Gastrointestinal Neonatal Surgery Department of Children’s Hospital affiliated Chongqing Medical University (a tertiary pediatric hospital and National Children’s Medical Center in China) during the period August 2019 to June 2020. NEC diagnosis was based on radiological evidence (i.e. pneumatosis intestinalis) and presence of one or more clinical fndings (i.e. abdominal distension, bilious/bloody aspirates, blood per rectum, abdominal tenderness, abdominal wall erythema/discoloration or abdominal mass) [19, 20]. Therefore cases of SIP were excluded. Furthermore, only patients who had complete laboratory data and not received fresh frozen plasma or platelet transfusions before 12 h of diagnosis were included in the final data set.

**Fig. 1:**
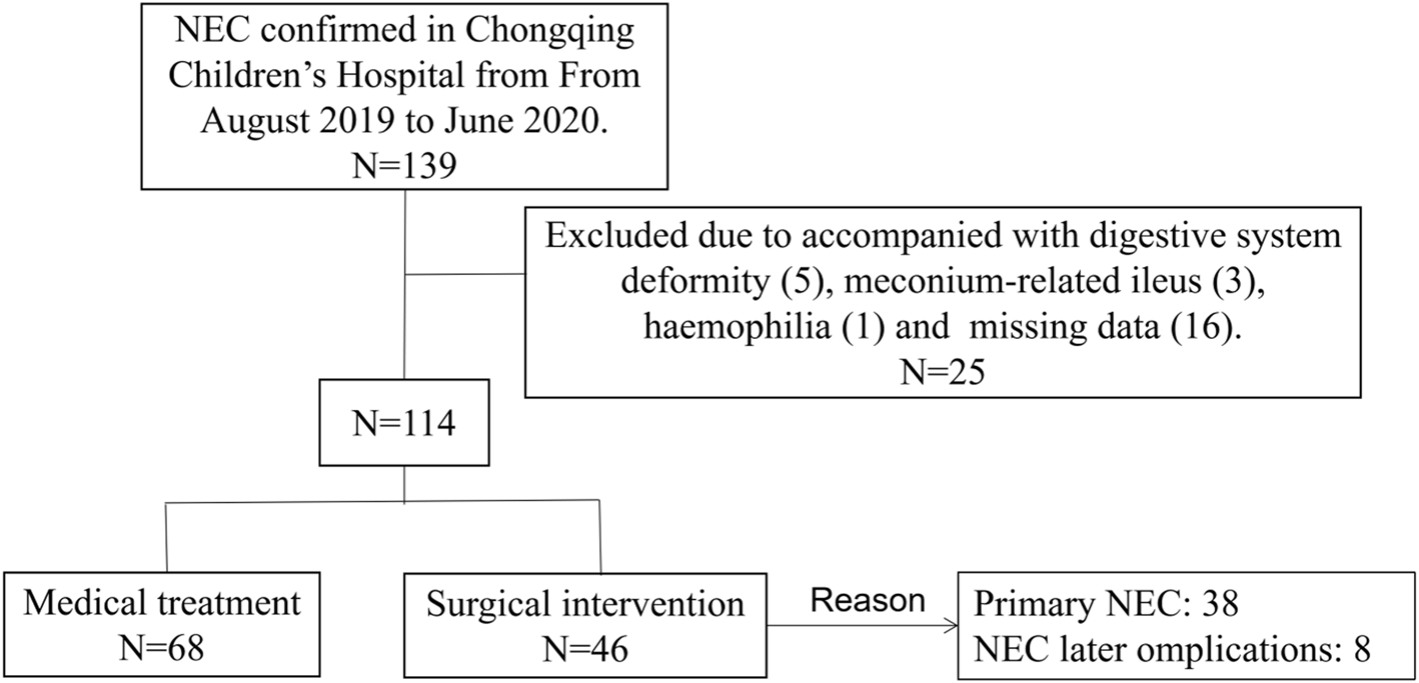
Flow chart of the study population

Patients were excluded if digestive system deformity that increase the risk of secondary NEC, like Hirschsprung’s disease, were identified (5 cases); they underwent operations for intestinal perforation due to meconium-related ileus (3 cases); the patient accompanied with haemophilia (1 case); or the clinical data was incomplete (16 cases). Figure 1 shows a flow diagram for the inclusion and exclusion of patients in this study.

In our hospital, neonatal surgery is performed by the permanent team (Yi Wang and Wei Liu). Indications for surgery were subsequent evidence of perforation (free intraperitoneal air on radiological examination), increasing abdominal distension, erythema, discoloration and tenderness despite maximal medical management and increasing ventilatory/inotropic support [2, 3]. Surgical intervention was defined as performance of exploratory laparotomy or placement of a peritoneal drain. To improve clinical practicability, the study also included the patients who received medical treatment for NEC but received surgery for later complications of NEC (eg, persistent intestinal obstruction or stricture) during 3-month follow-up period.

### Study design

The data collected for patients included demographic data: pregnancy, gender, gestational age, birth weight, five-minute Apgar score, postmenstrual age at NEC diagnosis, maternal age and vaginal delivery. Laboratory data, including general blood examinations and coagulation assessment, tested within 12 h after diagnosis of NEC were recorded. In our center, routine coagulation parameters (venous blood samples) were as follows: prothrombin time (PT), prothrombin time international normalized ratio (PT-INR), activated partial thromboplastin time (APTT), fibrinogen (Fib), thrombin time (TT), D-dimer (DD) and fibrinogendegradation products (FDP). In addition, we also collected data on red blood cell transfusions within 48 h before NEC diagnosis. The prognosis of interest was NEC-related death during hospitalization or within the 3-month follow-up period after discharge [21].

To date, the diagnostic criteria for coagulopathy in children remains controversial. According to relevant literature and the reference range of coagulation parameters in our hospital (PT: 9.8 ~ 13.3 [s]; PT-INR: 0.85 ~ 1.15; APTT: 25.0 ~ 35.4 [s]; Fib: 0.85 ~ 1.15 [g/L]; TT: 14.7 ~ 20.1 [s]; DD: < 0.73[mg/L]; FDP: < 5.0 [μg/dL]), coagulopathy was considerate when one of the disturbs were present: platelet count (PLT) less than 100 (× 10^9^/L), APTT superior to 45.4 s, and PT-INR superior to 1.3 [22–25].

### Statistical analysis

Excel software was used to data entry, Statistical Package for Social Sciences (SPSS) 22.0 softwares were used for statistical assessments.

Fisher’s exact test or chi-squared test, as appropriate, were used for comparison of categorical data, and Student’s t test or Mann Whitney-U test was used for numerical data. Tests for normality, collinearity and homoscedasticity were included for each quantitative variable in the analysis (data not shown). Univariable analysis was utilized to screen out the following 10 variables: WBC, HB, PLT, CRP, PCT, PT, PT-INR, APTT, Fib and DD, potentially affecting surgical NEC. Logistic regression was used to explore associations between potential independent variables and dependent variables taking into account clinically important and meaningful confounders [21]. Subsequently, receiver operating characteristics (ROC) analysis was obtained to identify the optimal cut-off values for coagulation parameters with sensitivity/specificity and to calculate the area under the ROC curves (AUC), respectively. In our statistical analysis, a *P* value < 0.05 was regarded as significant.

## Results

### General patients’ demographics

**Fig. 2:**
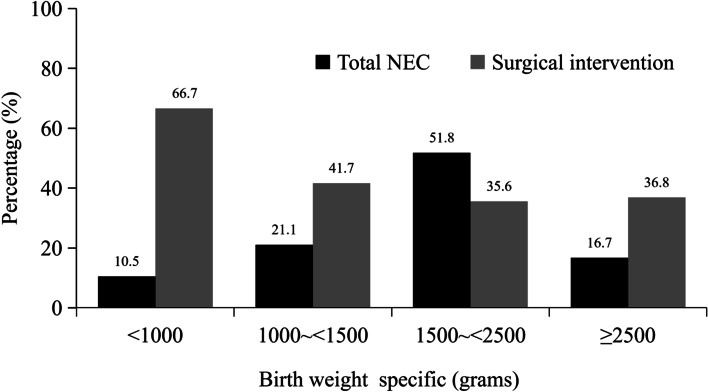
Showing birth weight specific cumulative distribution of study population

The entire number of patients met the the inclusion and exclusion criteria during the time frame of the study was 114: 61 females (53.5%) and 53 males (46.5%). Overall proportion of surgical intervention was 40.4% and 33.3% subjects received surgery for primary NEC. The patients with a median gestation at birth of 33 (range 25 ~ 42) weeks and birthweight of 1810 (range 480 ~ 3520) grams were all included. Majority (51.8%) of the cases belongs to low birth weight (1500 ~ 2500 g) (Fig. 2). The range of postmenstrual age at NEC diagnosis was 4 to 38 days, and majority (50.0%) of the cases diagnosed within 7 to 14 days.

### Group characteristics based on treatment methods for NEC

General patient demographics of these patients are presented in Table 1. For patients receiving medical treatment or surgical intervention, they were comparable in terms of basic information (gender, gestational age, birth weight and five-minute Apgar score), maternal factors (pregnancy, maternal age and vaginal delivery), postmenstrual age at NEC diagnosis, and proportion of cases receiving blood transfusion within 48 h before NEC diagnosis (all *P* > 0.05). For the results of general blood examinations (Table 2), patients receiving surgical intervention were significantly associated with an decreased WBC (median: 8.93 versus 10.19 [× 10^9^/L], *P* = 0.041), HB (mean: 133.20 versus 146.03 [g/L], *P* = 0.035), PLT (median: 163.0 versus 328.0 [× 10^9^/L], *P* < 0.001), and increased CRP (range distribution difference), PCT (median: 1.83 versus 0.33 [ug/L], *P* < 0.001). As for coagulation parameters, except TT and FDP (*P* > 0.05), differences of the remaining five (including PT, PT-INR, APTT, Fib and DD) between the two groups were statistically significant (all *P* < 0.05). PT (median: 18.5 versus 14.3 [s]), PT-INR (median: 1.12 versus 1.01), APTT (mean: 44.07 versus 36.27 [s]) and DD (median: 1.42 versus 1.12 [mg/L]) were all significantly higher in the surgical intervention group compared with medical treatment group, it may suggest that the patient receiving surgical intervention had worse coagulation function at the time of NEC diagnosis.

**Table 1:** General patient demographics in 114 neonates with NEC

Variables	Overall (*N* = 114)	Surgical intervention (*N* = 46)	Medical treatment (*N* = 68)	*P* value
Gender (n/%)^a^				0.596
Male	53 (46.5)	20 (43.5)	33 (48.5)	
Female	61 (53.5)	26 (56.5)	35 (51.5)	
Gestational age (weeks, n/%) ^a^				0.117
< 28	7 (6.1)	5 (10.9)	2 (2.9)	
28 ~ < 32	29 (25.4)	14 (30.4)	15 (22.2)	
32 ~ < 34	24 (21.1)	5 (10.9)	19 (27.9)	
34 ~ < 37	39 (34.2)	16 (34.8)	23 (33.8)	
≥ 37	15 (13.2)	6 (13.0)	9 (13.2)	
Birth weight (grams, n/%) ^a^				0.136
< 1000	12 (10.5)	8 (17.4)	4 (5.9)	
1000 ~ < 1500	25 (21.9)	12 (26.1)	13 (19.1)	
1500 ~ < 2500	58 (50.9)	19 (41.3)	39 (57.4)	
≥ 2500	19 (16.7)	7 (15.2)	12 (17.6)	
Pregnancy (n/%)^a^				0.163
Single	87 (76.3)	32 (69.4)	55 (80.9)	
Multiple	27 (23.7)	14 (30.4)	13 (19.1)	
Apgar at 5 min^b^	7.6 ± 1.3	7.8 ± 1.2	7.5 ± 1.3	0.241
Postmenstrual age at NEC diagnosis (days, n/%) ^a^				0.311
≤ 7	14 (12.3)	8 (17.4)	6 (8.8)	
> 7 ~ 14	57 (50.0)	20 (43.5)	37 (54.4)	
> 14	43 (37.7)	18 (39.1)	25 (36.8)	
Blood transfusion (n/%)				0.109
Yes	35 (30.7)	18 (39.1)	17 (25.0)	
No	79 (69.3)	28 (60.9)	51 (75.0)	
Maternal age^b^	29.7 ± 4.6	30.1 ± 4.5	29.4 ± 4.6	0.422
Vaginal delivery (n/%)				0.651
Yes	45 (39.5)	17 (37.0)	28 (41.2)	
No	69 (60.5)	29 (63.0)	40 (58.8)	

^a^Chi-square test

^b^Values are presented as mean ± standard deviation and used Student’s t test

**Table 2:** Hematological comparison between medical treatment and surgical intervention

Blood test parameters	Overall (*N* = 114)	Surgical intervention (N = 46)	Medical treatment (*N* = 68)	*P* value
General blood examination				
WBC (× 10^9^/L)^a^	9.64 (6.80,13.06)	8.93 (5.60,11.16)	10.19 (7.18,13.68)	0.041
HB (g/L)^c^	140.85 ± 32.02	133.20 ± 28.31	146.03 ± 33.52	0.035
PLT (× 10^9^/L)^a^	277.0 (157.8,406.5)	163.0 (85.5,305.8)	328.0 (256.3,433.8)	< 0.001
CRP (mg/L)^b^				0.026
< 8	68 (59.6)	21(45.7)	47 (69.1)	
8 ~ 20	18 (15.8)	8 (17.4)	10 (14.7)	
> 20 ~ 50	19 (16.7)	10 (21.7)	9 (13.2)	
> 50	9 (7.9)	7 (15.2)	2 (2.9)	
PCT (ug/L)^a^	0.67 (0.15,4.39)	1.83 (0.58,12.75)	0.33 (0.12,2.65)	< 0.001
Coagulation assessment				
PT (s)^a^	15.6 (13.3,19.5)	18.5 (15.6,20.4)	14.3 (13.1,16.5)	< 0.001
PT-INR^a^	1.08 (0.92,1.22)	1.12 (1.01,1.34)	1.01 (0.92,1.18)	0.008
APTT (s)^c^	39.42 ± 7.02	44.07 ± 7.07	36.27 ± 4.97	< 0.001
Fib (g/L)^a^	1.22 (1.06,1.51)	1.11 (1.00,1.31)	1.34 (1.13,1.62)	< 0.001
TT (s)^a^	20.4 (17.9,24.9)	20.7 (17.7,25.7)	20.1 (17.9,24.5)	0.540
DD (mg/L)^a^	1.22 (0.91,5.19)	1.42 (1.07,21.59)	1.12 (0.79,1.40)	< 0.001
FDP (μg/dL)^a^	7.76 (4.73,11.37)	7.69 (4.65,11.22)	7.80 (4.58,11.80)	0.887

*WBC* White blood cell count, *HB* Hemoglobin, *PLT* Platelet count, *CRP* C-reactive Protein, *PCT* Procalcitonin, *PT* Prothrombin Time, *PT-INR* PT International Normalized Ratio, *APTT* Activated Partial Thromboplastin Time, *Fib* Fibrinogen, *TT* Thrombin Time, *DD* D-dimer, *FDP* Fibrinogendegradation Products

^a^Values are presented as medians (IQR) and used Mann–Whitney U test;

^b^Chi-square test;

^c^Values are presented as mean ± standard deviation and used Student’s t test

### Predictive value of coagulation parameters for surgical NEC

Univariable analysis was utilized to determine the effects of potential factors on surgical NEC. Multivariate regression analysis is conducive to balancing the interactions between variables and comparable so that non-random grouping data can be used to study the relationship between trial factors and dependent variable, and obtain more reliable research results. So, the following 10 variables: WBC, HB, PLT, CRP, PCT, PT, PT-INR, APTT, Fib and DD, were included in the stepwise multivariate logistic regression model, and independent predictors were identified. Significant independent predictors were (Table 3): PT (OR = 1.289, 95% CI: 1.072 ~ 1.549, *P* = 0.007), PT-INR (OR = 83.048, 95% CI: 4.498 ~ 133.325, *P* = 0.003), APTT (OR = 1.250, 95% CI: 1.119 ~ 1.398, P < 0.001), Fib (OR = 0.170, 95% CI: 0.035 ~ 0.818, *P* = 0.027), and PLT (OR = 0.995, 95% CI: 0.990 ~ 0.999, *P* = 0.029). According to the values of ORs, it is suggested that the change of PT-INR had the greatest influence on the risk of surgical intervention. An increase in the PT-INR of 1 unit resulted in an increase in the risk of surgery by 83.048 times.

**Table 3:** Multivariate Logistic regression analysis for the surgical NEC

Variables	β	OR	95% CI	*P* value
PT (s)	0.254	1.289	1.072 ~ 1.549	0.007
PT-INR	4.419	83.048	4.498 ~ 133.325	0.003
APTT (s)	0.223	1.250	1.119 ~ 1.398	< 0.001
Fib (g/L)	-1.774	0.170	0.035 ~ 0.818	0.027
PLT (× 10^9^/L)	-0.005	0.995	0.990 ~ 0.999	0.029

*β* Regression coefficient, *OR* Odds Ratio, *95% CI* 95% Confidence Interval, *NEC* Necrotizing Enterocolitis, *PT* Prothrombin Time, *PT-INR* PT International Normalized Ratio, *APTT* Activated Partial Thromboplastin Time, *Fib* Fibrinogen, *PLT* Platelet count

**Fig. 3:**
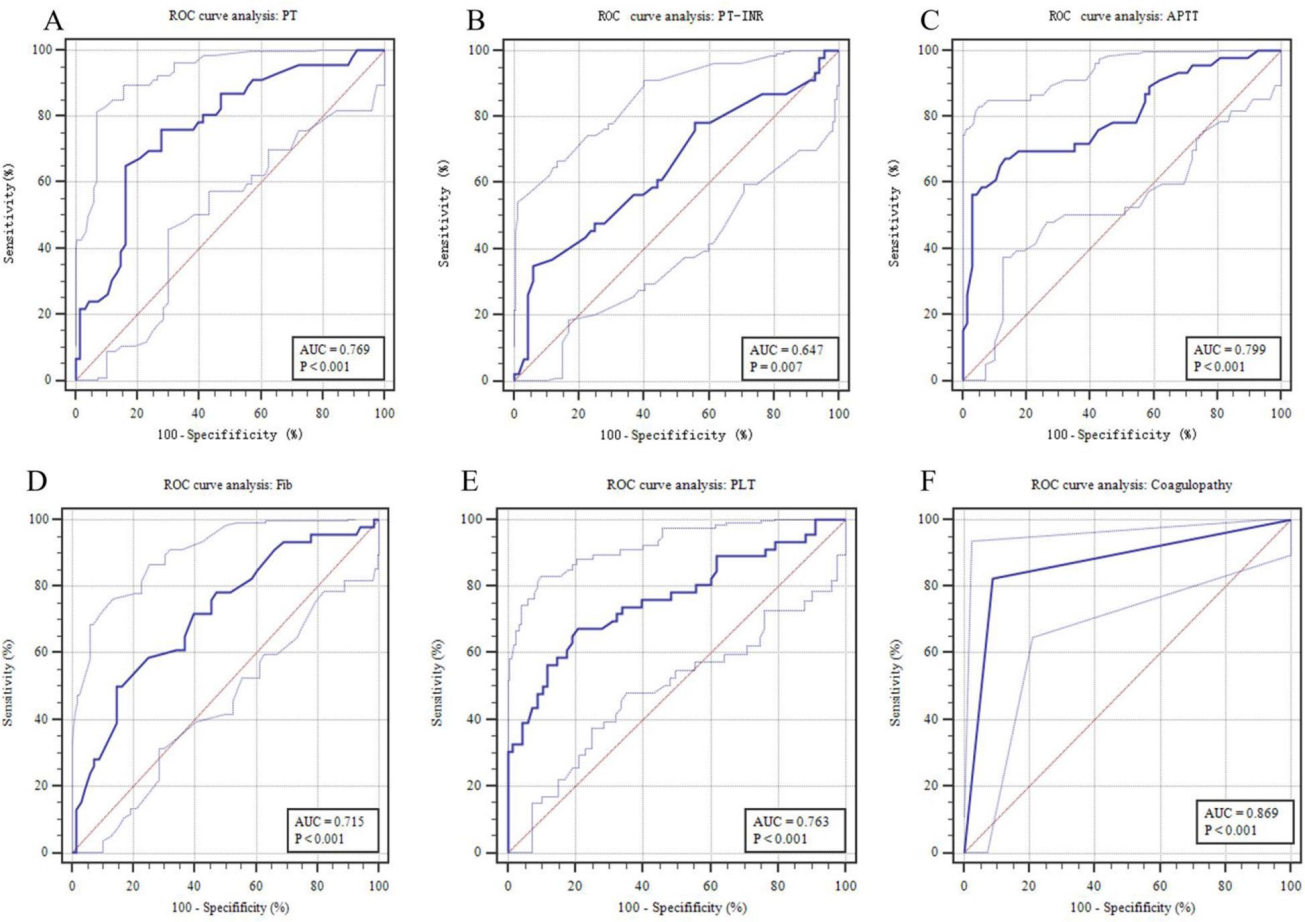
Predictive assessment of coagulation parameters for patients receiving surgery with ROC curve analysis. ROC, receiver operating characteristics; AUC, area under the curve

**Fig. 4:**
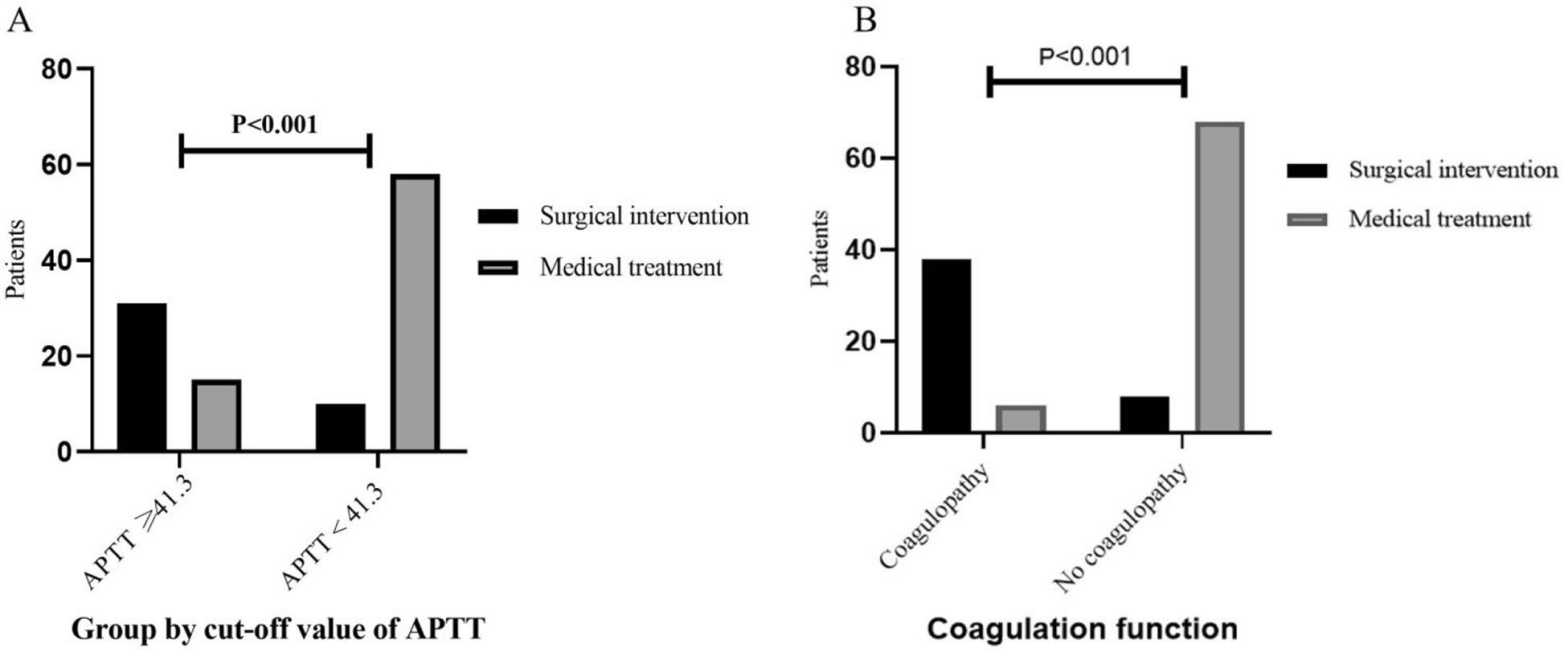
The number of patients intervened with surgery in different subgroups

ROC curve analysis of the significant independent predictors was shown in Fig. 3 (A-E). The AUCs of PT, PT-INR, APTT, Fib and PLT were 0.769, 0.647, 0.799, 0.715 and 0.763, respectively. The APTT showed a clearly better predictive performance for identifying the patients who received surgical intervention than PT, PT-INR, Fib and PLT. When the APTT was 41.3 s, the Youden index was the largest (0.54). The predictive values of APTT were 67.39% sensitivity, 86.76% specificity, 77.50% positive predictive value (PPV) and 79.73% negative predictive value (NPV). Independent variables were used to evaluate the predictive value of surgical NEC, and predictive parameter (i.e. sensitivity, specificity, PPV and NPV) corresponding to the optimal cut-off values were shown in Table 4. According to the optimal cut-off value of APTT (41.3 s), patients with the APTT of 41.3 or greater at the time of NEC diagnosis were considered to be more likely to received surgery. It was found in 67.4% of patients received surgery and in 14.7% of those received medical treatment (*P* < 0.001, Fig. 4A). Compared with a APTT < 41.3 s, patients with APTT ≥ 41.3 s had a 11.987 times higher chance of receiving surgical intervention (95% CI: 4.819 ~ 29.816).

**Table 4:** Predictive values of coagulation parameters for surgical NEC

Variables	The best cut-off level	Youden index (largest)	Sensitivity (%)	Specifificity (%)	PPV (%)	NPV (%)
PT (s)	16.7	0.49	65.22	83.82	73.17	78.08
PT-INR	1.23	0.29	34.78	94.20	80.00	68.09
APTT (s)	41.3	0.54	67.39	86.76	77.50	79.73
Fib (g/L)	1.09	0.35	50.00	85.29	65.52	68.24
PLT (× 10^9^/L)	252	0.47	67.39	79.41	69.77	77.46

*NEC* Necrotizing Enterocolitis, *PPV* Positive Predictive Value, *NPV* Negative Predictive Value, PPV and NPV were calculated for the best cut-off levels. *PT* Prothrombin Time, *PT-INR* PT International Normalized Ratio, *APTT* Activated Partial Thromboplastin Time, *Fib* Fibrinogen, *PLT* Platelet Count

### Predictive value of coagulopathy

From the multivariate logistic regression analysis results, we found that coagulation parameters, including PT, PT-INR, APTT, Fib and PLT, were independent predictors of the NEC receiving surgery. It presume that patient who received surgical intervention had worse coagulation function than those treated conservatively. To evaluate whether the coagulopathy (definition described above) could accurately predict the patients who need surgical intervention, AUC of ROC was used to quantify the predictive value. By definition, we found coagulopathy in 44 of the 114 patients (38.6%). At the time of NEC diagnosis, coagulopathy was found in 82.6% of surgical intervention group and in 8.8% of medical treatment group (*P* < 0.001, Fig. 4B). For surgical intervention, the AUC of coagulopathy was 0.869 (95% CI: 0.794 ~ 0.944, *P* < 0.001, Fig. 3F), with 82.61% sensitivity, 91.18% specificity, 86.36% PPV and 88.57% NPV. Compared with no coagulopathy, the coagulopathy had a 49.083 times higher chance of receiving surgical intervention (95% CI: 15.810 ~ 152.387). In addition, AUCs comparison showed that coagulopathy outperformed APTT in predicting surgical NEC (95% CI: 0.236 ~ 0.173, *P* = 0.001).

### Both surgical intervention and coagulopathy associated with mortality

**Fig. 5:**
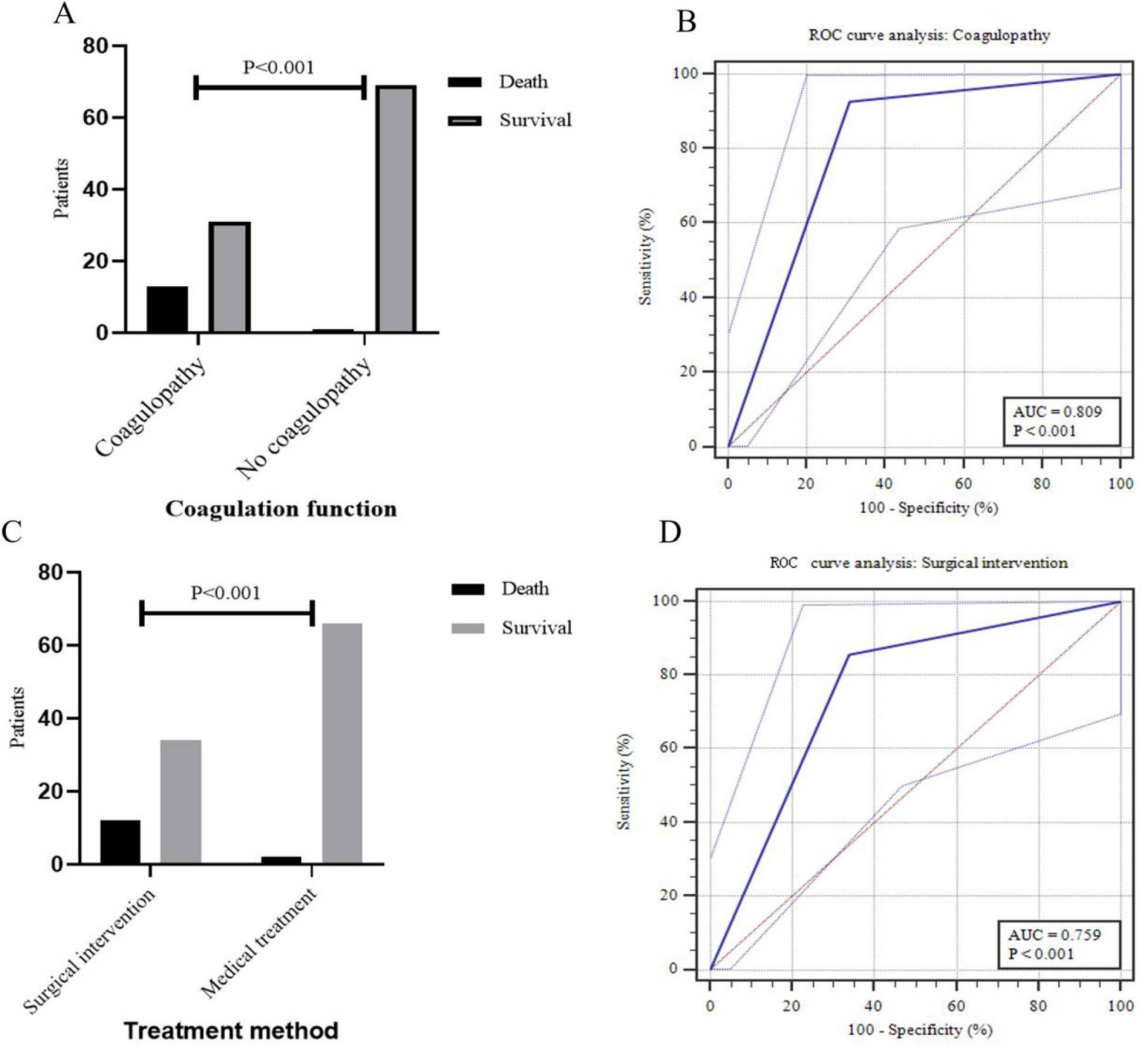
Predictive analysis of prognosis for surgical intervention and coagulopathy. ROC, receiver operating characteristics; AUC, area under the curve

There were 14 (12.3%) deaths during hospitalization or within the 3-month follow-up period after discharge in this cohort. We found that 92.9% (13/14) of patients who died had coagulopathy at the time of NEC diagnosis, while the incidence was 30.0% (31/100) in patients who survived (χ^2^ = 19.827, *P* < 0.001, Fig. 5A). And the AUC for coagulopathy to predict mortality was 0.809 (95% CI: 0.725 ~ 0.877, *P* < 0.001, Fig. 5B), with 92.86% sensitivity, 69.0% specificity, 29.55% PPV and 98.57% NPV. Mortality rate was significantly higher in surgical intervention group than in conservative treatment group (rate: 26.1% versus 2.9%, χ^2^ = 13.645, *P* < 0.001, Fig. 5C). For mortality, the AUC of surgical intervention was 0.759 (95% CI: 0.669 ~ 0.834, *P* < 0.001, Fig. 5D), with 85.71% sensitivity, 66.0% specificity, 26.09% PPV and 97.06% NPV. The results suggested that coagulopathy at the time of NEC diagnosis was not only early predictor of mortality, but also had accurate predictive validity.

## Discussion

There have been some studies on the prognosis of NEC, but very few reports have focused on the serological indicator for early prediction of surgical NEC and prognosis; thus, we designed this study [26–28]. In this retrospective study, we found that PT, PT-INR, APTT, Fib and PLT at the time of NEC diagnosis were independently associated with surgical NEC. Regarding prediction, the APTT could identify patients at high risk for surgical NEC, with sensitivity of 67.39% and specificity of 86.76%, better than that of other serological parameters. Based on multivariate regression results, we hypothesized that coagulopathy, as defined by coagulation parameters ranges, may be more conducive to early prediction of surgical NEC and prognosis. Upon statistical analysis, coagulopathy showed fair predictive accuracy, and might be used to facilitate the development of therapeutic strategies for NEC. Hence, research on the risk factors for surgical NEC and prognosis must focus on high-risk populations for coagulopathy by assessing the balance between potential benefits and harms.

Recent researches reported that more than 30% of neonates with NEC may require surgical intervention, and mortality rate among surgical NEC cases is estimated to be 20% ~ 30% [3, 21]. But in this cohort, we found that overall proportion of surgical intervention was 40.4% (33.3% cases received surgery for primary NEC and 7.1% for later complications), the rate of surgical intervention was higher than most reports. Probably due to the fact that our hospital is the largest tertiary pediatric referral centre in southwest China (National Clinical Medical Research Centre for Child Health and Disease), most of the patients admitted were in relatively serious condition. However, it should be noted that the overall mortality rate was 12.3%, including 26.1% following surgical intervention and 2.9% receiving medical treatment. This indicates that the prognosis of NEC following surgery is still poor and of note is unchanged in recent years despite improvements in neonatal care and surgical technique [3, 29]. Therefore, if we can reduce the mortality of surgical NEC, then it is beneficial to improve the overall outcome of NEC and future research should focus on this direction.

Ample evidence has demonstrated that a wide-ranging cross-talk between inflammation and coagulation, which is probably assisted in identifying the severity of inflammation and provided potential therapeutic targets [30–32]. Recent studies have shown that coagulation dysfunction were often present in critically patients with COVID-19, and the prognosis was worse in patients with coagulopathy [10–13]. This phenomenon has also been found in sepsis [30]. Inflammation not only leads to initiation and propagation of coagulation activity, but coagulation dysfunction also markedly influences the progression of inflammation [5]. Molecular mechanisms of inflammation-induced coagulation dysfunction have been reported, and pro-inflammatory cells (ie, macrophages and neutrophils) and secreted chemokines (ie, tumor necrosis factor-ɑ and interleukin-1/6) can activate the coagulation system and downregulate crucial physiological anticoagulant mechanisms [5]. But when the procoagulant stimulus is sufficiently severe and overcomes the natural anticoagulant mechanisms of coagulation, disseminated intravascular coagulation (DIC) may occur [33]. Hence, adjunctive therapeutic strategies for coagulation impairment, including anticoagulants and restoration of physiological anticoagulant mechanisms, can help reduce the severity of inflammation and even improve the overall prognosis [31]. Coagulation parameters are the indirect reflection of coagulation status and have been reported clinically to make preliminarily judgements about condition and prognosis, so as to develop timely coping strategies [7, 25, 34].

NEC, an inflammatory bowel necrosis of neonates, is characterized by severe inflammatory response in the gut and systemic, intestinal ischemia, thrombocytopenia, and DIC. Hutter et al. noted signs of DIC in 35% of neonates with NEC [35]. Many of these patients showed the changes of coagulation parameters as follows: plasma Fib and PLT levels decreased, FDP tested positive, and APTT elevated [36]. These data are similar to this respective cohorts. Research has reported that the gene expression of coagulation- and anticoagulation-related proteins in NEC had significant alterations, and these genes were associated with overall procoagulant status, reduction in fibrinolysis and worse endothelial regeneration [15, 37]. This could be the main reason for bowel inflammation progression. Coagulopathy leads to progressive aggravation of intestinal damage, including mesenteric thrombosis, patchy intestinal ischemia and perforation. Giuliani et al. suggested that coagulation status could serve as a potential biomarker for disease progression in NEC [15, 37]. It is worth noting that the case sample taken in the above studies included advanced NEC (Bell’s stage III), and there is a lack of studies that describe coagulation function at the time of NEC diagnosis separately.

In our study, we found coagulopathy in 38.6% of the patients at the time of NEC diagnosis, and patient who received surgical intervention had worse coagulation function than those treated conservatively. Of the coagulation parameters, APTT showed a clearly better predictive performance (67.39% sensitivity and 86.76% specificity) for identifying the patients who received surgical intervention than PT, PT-INR, Fib and PLT. Three parameters defining coagulopathy, including PLT, APTT and PT-INR, were shown to be independent predictors of surgical NEC. Therefore, we hypothesized that coagulopathy could be more accurate in early prediction of surgical NEC. AUCs comparison showed that coagulopathy was more more predictive than APTT. It can be used as a reference to help us communicate possibility of receiving surgery with family members fully, as well as target limited medical resources for those at the highest risk. In addition, for medical institution that does not perform neonatal surgery, early identification of patient at high risk for surgery is more conducive to timely referral. Interestingly, the results showed that coagulopathy could increase the risk of death and be an effective predictor. If early coagulation assessment can detect and correct coagulopathy timely, it may be beneficial to reduce the mortality of NEC. This study not only describe the independent risk factors for surgical NEC, but confirm that coagulopathy can effectively predict surgical NEC and prognosis. To our knowledge, we were the first to show early coagulopathy for NEC with two uses.

The retrospective analysis was conducted in one hospital and the cohort was relatively small, further research with larger prospective and multi-centre cohorts is necessary to validate the usefulness of the coagulation parameters for predicting surgical NEC and prognosis. Therefore, unreported confounders could not necessarily be ruled out, which may lead to the potential result bias. Secondly, considering that our hospital is the largest tertiary referral center for children in southwest China, the neonates in this cohort were in relatively seriously condition. Finally, not all patients routinely received coagulation assessment at the time of NEC diagnosis, and we excluded this group of patients. It is important to note that the exclusion of patients with missing data inevitably carry the risk of selection bias in this retrospective study. We also recommend that coagulation function should be closely monitored in neonates at high risk of NEC in future clinical practice.

## Conclusion

To our knowledge, this is the first study analyzing the relationship between coagulation parameters at the time of NEC diagnosis and surgical intervention, prognosis. We identified that PT, PT-INR, APTT, Fib and PLT were independently associated with surgical NEC, and APTT could identify patients at high risk for surgical NEC, better than that of other serological markers. Further analysis revealed that coagulopathy could be more conducive to early prediction of surgical NEC and prognosis. This finding may be utilized to triage neonates for transfer to specialist neonatal surgical centers, which would optimize surgical cot utilization, and provided reference to guide parental counselling.

## Data Availability

The datasets generated and analysed during the current study are not publicly available due the ongoing analysis in other directions but are available from the corresponding author on reasonable request.

## Abbreviations

NEC: Necrotizing enterocolitis
PT: Prothrombin time
PT-INR: PT international normalized ratio
APTT: Activated partial thromboplastin time
Fib: Fibrinogen
TT: Thrombin time
DD: D-dimer
FDP: Fibrinogendegradation products
AUC: Area under the curve
